# Supplementary material for: Factors Associated with Refractive Prediction Error after Phacotrabeculectomy
Source: J Clin Med. 2023 Sep 1;12(17):5706. doi: 10.3390/jcm12175706 (PMC10488334; doi:10.3390/jcm12175706)
Supplement: Supplementary file 1 [file jcm-12-05706-s001.zip › Supplemental table.pdf]

Table S1. Factors associated with absolute prediction error

| | Univariable analysis | | | Multivariable analysis (Model 1)[a] | | | Multivariable analysis (Model 2)[a] | | |
|---|---|---|---|---|---|---|---|---|---|
| | Coefficient | 95% CI | *P* | Coefficient | 95% CI | *P* | Coefficient | 95% CI | *P* |
| Age, years | -0.001 | -0.007, 0.005 | 0.765 | | | | | | |
| Female (vs. male sex) | -0.062 | -0.190, 0.066 | 0.341 | | | | | | |
| **AL, mm** | **0.046** | **0.006, 0.087** | **0.026** | **0.050** | **0.010, 0.009** | **0.015** | **0.047** | **0.008, 0.087** | **0.020** |
| ACD, mm | 0.031 | -0.086, 0.148 | 0.598 | | | | | | |
| Average K, Diopter | -0.009 | -0.051, 0.034 | 0.694 | | | | | | |
| Four-haptic IOL (vs. two-haptic IOL) | 0.056 | -0.094, 0.206 | 0.460 | | | | | | |
| **Preoperative IOP, mmHg** | **0.005** | **-0.000, 0.011** | **0.062** | **0.006** | **0.000, 0.011** | **0.035** | | | |
| **Phacotrabeculectomy (vs. control group)** | **0.135** | **0.011, 0.258** | **0.033** | | | | **0.138** | **0.018, 0.259** | **0.025** |

CI = confidence interval; AL = axial length; ACD = anterior chamber depth; IOL = intraocular lens; IOP = intraocular pressure

[a]Variables with *P*<0.10 in the univariable analysis were included in the subsequent multivariable analysis. Owing to multicollinearity between the groups and preoperative IOP, two multivariable analysis models were constructed. The result of multivariable analysis with backward elimination was equivalent to that of Model 1. Statistically significant values (*P*<0.05) are shown in bold.

Table S2. Risk factors for hyperopic prediction error

| | Univariable analysis | | | Multivariable analysis (Model 1)[a] | | | Multivariable analysis (Model 2)[a] | | |
|---|---|---|---|---|---|---|---|---|---|
| | OR | 95% CI | *P* | OR | 95% CI | *P* | OR | 95% CI | *P* |
| Age, years | 1.030 | 0.982, 1.081 | 0.220 | | | | | | |
| Female (vs. male sex) | 1.784 | 0.655, 4.856 | 0.257 | | | | | | |
| AL, mm | 0.570 | 0.341, 0.953 | 0.032 | 0.626 | 0.372, 1.052 | 0.077 | | | |
| **ACD, mm** | **0.180** | **0.062, 0.517** | **0.001** | | | | **0.256** | **0.085, 0.773** | **0.016** |
| Average K, Diopter | 1.186 | 0.862, 1.631 | 0.296 | | | | | | |
| Four-haptic IOL (vs. two-haptic IOL) | 0.763 | 0.540, 1.079 | 0.126 | | | | | | |
| **Preoperative IOP, mmHg** | **1.066** | **1.022, 1.111** | **0.003** | **1.065** | **1.004, 1.129** | **0.037** | 1.050 | 0.989, 1.115 | 0.110 |
| Phacotrabeculectomy (vs. control group) | 0.821 | -0.153, 1.795 | 0.099 | 0.842 | 0.205, 3.453 | 0.811 | 0.813 | 0.197, 3.357 | 0.775 |

CI = confidence interval; AL = axial length; ACD = anterior chamber depth; IOL = intraocular lens; IOP = intraocular pressure

[a]Variables with *P*<0.10 in the univariable analysis were included in the subsequent multivariable analysis. Because of multicollinearity between AL and ACD, two models were constructed. The result of multivariable analysis with backward elimination was equivalent to that of Model 2. Statistically significant values (*P*<0.05) are shown in bold.

Table S3. Factors associated with prediction error after phacotrabeculectomy

| | Univariable analysis | | | Multivariable analysis (Model 1)[a] | | | Multivariable analysis (Model 2)[a] | | |
|---|---|---|---|---|---|---|---|---|---|
| | Coefficient | 95% CI | *P* | Coefficient | 95% CI | *P* | Coefficient | 95% CI | *P* |
| Age, years | 0.007 | -0.008, 0.023 | 0.359 | | | | | | |
| Female (vs. male sex) | 0.402 | 0.068, 0.736 | 0.019 | 0.199 | -0.152, 0.549 | 0.259 | 0.310 | -0.042, 0.663 | 0.083 |
| **AL, mm** | **-0.195** | **-0.297, -0.094** | **<0.001** | **-0.193** | **-0.325, -0.060** | **0.005** | | | |
| **ACD, mm** | **-0.418** | **-0.720, -0.115** | **0.008** | | | | **-0.491** | **-0.967, -0.014** | **0.044** |
| Average K, Diopter | 0.064 | -0.043, 0.171 | 0.236 | | | | | | |
| Four-haptic IOL (vs. two-haptic IOL) | 0.054 | -0.513, 0.622 | 0.848 | | | | | | |
| Preoperative IOP, mmHg | 0.012 | -0.003, 0.027 | 0.109 | | | | | | |
| ACG (vs. OAG) | 0.294 | -0.049, 0.638 | 0.091 | -0.132 | -0.524, 0.261 | 0.502 | -0.241 | -0.767, 0.285 | 0.361 |

CI = confidence interval; AL = axial length; ACD = anterior chamber depth; IOL = intraocular lens; IOP = intraocular pressure; ACG = angle-closure glaucoma; OAG = open-angle glaucoma

[a]Variables with *P*<0.10 in the univariable analysis were included in the subsequent multivariable analysis. Because of multicollinearity between AL and ACD, two models were constructed. Multivariable analysis with backward elimination left only AL as the associative factor. Statistically significant values (*P*<0.05) are shown in bold.
